# Delafloxacin *In Vitro* Broth Microdilution and Disk Diffusion Antimicrobial Susceptibility Testing Guidelines: Susceptibility Breakpoint Criteria and Quality Control Ranges for an Expanded-Spectrum Anionic Fluoroquinolone

**DOI:** 10.1128/JCM.00339-18

**Published:** 2018-07-26

**Authors:** M. A. Pfaller, R. K. Flamm, S. P. McCurdy, C. M. Pillar, D. Shortridge, R. N. Jones

**Affiliations:** a JMI Laboratories, North Liberty, Iowa, USA; b Melinta Therapeutics, New Haven, Connecticut, USA; c Micromyx, LLC, Kalamazoo, Michigan, USA; Boston Children's Hospital

**Keywords:** delafloxacin, fluoroquinolones, broth microdilution, disk diffusion, quality control, susceptible breakpoints, susceptibility, testing criteria

## Abstract

Delafloxacin, a recently approved anionic fluoroquinolone, was tested within an international resistance surveillance program. The *in vitro* susceptibilities of 7,914 indicated pathogens causing acute bacterial skin and skin structure infections (ABSSSI) were determined using Clinical and Laboratory Standards Institute (CLSI) broth microdilution MIC testing methods. The U.S. Food and Drug Administration (FDA) susceptibility testing breakpoints and quality control ranges for routine broth microdilution and disk diffusion methods were confirmed. The delafloxacin MIC_50/90_ (% susceptibility) results were as follows: Staphylococcus aureus, including methicillin-resistant S. aureus (MRSA), 0.008/0.25 μg/ml (92.8%); Staphylococcus lugdunensis, 0.016/0.03 μg/ml (99.3%); Streptococcus pyogenes, 0.016/0.03 μg/ml (100.0%); Streptococcus anginosus group, 0.008/0.016 μg/ml (100.0%); Enterococcus faecalis, 0.12/1 μg/ml (66.2%); and Enterobacteriaceae, 0.12/4 μg/ml (69.5%). The FDA clinical breakpoints were used to assess intermethod test agreement between delafloxacin MIC and disk diffusion methods for the indicated pathogens. The intermethod susceptibility test categorical agreement for delafloxacin was acceptable, with only 0.4% very major, false-susceptible errors among S. aureus strains. Across all FDA-indicated species, the selected breakpoints produced only 0.0 to 1.7% rates of serious (very major and major errors) intermethod error. Quality control ranges for these standardized delafloxacin susceptibility test methods were calculated from three multilaboratory (12 total sites) studies for six control organisms. In conclusion, the application of FDA MIC breakpoints for delafloxacin against contemporary (2014 to 2016) isolates of ABSSSI pathogens provides additional support for the use of delafloxacin in the treatment of adults with ABSSSI. Delafloxacin MIC and disk diffusion susceptibility testing methods have been standardized for clinical application, achieving high intermethod categorical agreement.

## Abstract (precis)

Delafloxacin, a recently approved anionic fluoroquinolone, was tested within an international resistance surveillance program. The *in vitro* susceptibilities of 7,914 indicated pathogens causing acute bacterial skin and skin structure infections (ABSSSI) were determined using Clinical and Laboratory Standards Institute (CLSI) broth microdilution MIC testing methods.

## INTRODUCTION

The fluoroquinolone (FQ) class of antimicrobial agents are indicated for empirical and directed therapy of susceptible pathogens implicated in urinary tract infections (UTI), respiratory tract infections (RTI), acute bacterial skin and skin structure infections (ABSSSI), and intra-abdominal infections (1–4). Extensive use and misuse of these drugs led to the emergence and spread of FQ-resistant (FQ-R) strains of virtually all species of bacteria shortly after introducing these compounds into clinical practice (1, 2, 5). Surveillance studies have documented increased FQ resistance rates affecting patient management, which necessitates a change in treatment guidelines for UTI, intra-abdominal infections, and ABSSSI (2, 5–7). Indeed, concerns regarding FQ resistance have precluded using these agents in treating sepsis (8) and sexually transmitted diseases (9). Efforts to combat this resistance within the FQ class have focused on developing candidates with improved activity against FQ-R bacteria and providing a lower potential for bacterial resistance development (1, 2, 5, 10–13).

Delafloxacin (formerly ABT-492) is an anionic fluoroquinolone with documented efficacy in phase 2 trials of RTI and ABSSSI, as well as in recently completed phase 3 trials for the treatment of patients with ABSSSI (1, 14, 15). Delafloxacin was approved by the U.S. Food and Drug Administration (FDA) in June 2017 for the treatment of adults with ABSSSI due to designated susceptible pathogens (16). The FDA-indicated pathogens include Staphylococcus aureus (including methicillin-resistant and -susceptible isolates), Staphylococcus haemolyticus, Staphylococcus lugdunensis, Streptococcus pyogenes, Streptococcus agalactiae, Streptococcus anginosus group (including S. anginosus, S. intermedius, and S. constellatus), Enterococcus faecalis, Escherichia coli, Klebsiella pneumoniae, Enterobacter cloacae, and Pseudomonas aeruginosa (16).

Despite national and international efforts to combat antimicrobial resistance, it is notable that clinical microbiology laboratories struggle to generate accurate and actionable antimicrobial susceptibility test (AST) reports (17). Given that the majority of laboratories in the United States use automated AST systems (Vitek 2 [bioMérieux], MicroScan [Beckman Coulter], BD Phoenix [Becton Dickinson]) exclusively for routine testing, the ability to respond to the introduction of new antimicrobial agents and to adapt to emerging resistance mechanisms as well as to changes in interpretive criteria (breakpoints) are compromised by lag times between the approval of new agents or updates in clinical breakpoints and the clearance on commercial automated AST devices (17, 18). In an effort to circumvent these barriers to providing AST data in a timely manner, laboratories may resort to using surrogate agents that may predict the susceptibility of the organism of interest to the newly introduced agent or to using manual AST methods, such as disk diffusion (DD) (17). The DD method advantages are the simplicity of the test, which does not require any special equipment, the provision of categorical results easily interpreted by clinicians, low cost, and flexibility in selecting drug disks for testing (18).

The recent regulatory approval of delafloxacin to treat ABSSSI (16) and the fact that there is no reliable surrogate among the FQs to predict the activity of delafloxacin against target pathogens mean that laboratories must adopt a nonautomated method, such as DD (delafloxacin disks are available in the United States from Hardy Diagnostics, Santa Maria, CA, USA), to provide AST information for this new broader-spectrum agent. The present study was designed to (i) assess the *in vitro* antibacterial activity of delafloxacin against contemporary (2014 to 2016) ABSSSI pathogens from the U.S. and European (EU) medical centers using FDA MIC interpretive criteria; (ii) determine the intermethod agreement between delafloxacin MIC and DD zone diameter values based on the FDA interpretive criteria; (iii) validate the FDA MIC quality control ranges for six aerobic quality control (QC) organisms.

## MATERIALS AND METHODS

### Study designs.

International surveillance studies (involving 5,187 U.S. isolates and 2,727 EU isolates from 22 countries) monitored delafloxacin activity by reference BMD methods (19) during 2014 to 2016 via a central laboratory protocol (JMI Laboratories, North Liberty, IA). These 7,914 organisms from ABSSSI were collected as part of the SENTRY Antimicrobial Surveillance Program, and results were focused on the species listed in the delafloxacin product package insert (Table 1) (16). Key comparison agents were also tested (levofloxacin, oxacillin) to characterize drug-resistant subpopulations using breakpoint interpretive criteria of the CLSI and the European Committee on Antimicrobial Susceptibility Testing (20, 21). QC was ensured by testing with ATCC reference strains and using performance guidelines found in published documents (16, 20).

Delafloxacin BMD MIC and 5-μg DD zone diameter comparison investigations utilized reference methods (19, 22), FDA MIC and DD breakpoints (18; see also Table 2), and accepted intermethod analyses (23). Organisms of indicated species or groups were analyzed separately, attempting to achieve minimal intermethod categorical errors. The organisms studied were from clinical trial samples (*n* = 2,214) and preclinical microbiology investigations (*n* = 1,500). All isolates were identified by standard biochemical tests or matrix-assisted laser desorption ionization–time of flight mass spectrometry (Bruker, Billerica, MA).

Determinations of QC limits for standardized tests (19, 22) were guided by a multilaboratory study design of the CLSI (23). For the delafloxacin MIC and DD methods, three separate studies were completed (two for zone diameter limits and a single MIC trial).

In each trial, a minimum of seven laboratories contributed test results for the studied ATCC QC strains: S. aureus ATCC 25922 (DD only) and ATCC 29213 (MIC only), S. pneumoniae ATCC 49619, E. faecalis ATCC 29212 (MIC only), E. coli ATCC 25922, and P. aeruginosa ATCC 27853. These investigations used at least three medium lots from two or more manufacturers (BD, Remel, Tekova) and two disk lots (MAST). Ranges were calculated by methods found in CLSI M23 (23) or by the RangeFinder program (24).

## RESULTS

### Antimicrobial activity of delafloxacin.

The reference broth microdilution MIC distributions for the FDA-indicated organisms or organism groups (7,914 isolates) from global (U.S. and EU) surveillance in 2014 to 2016 are shown in Table 1. The MIC_90_ values for U.S. and EU isolates of Gram-positive cocci (GPC) were within ± 1 log_2_ dilution step for each organism group except MSSA (MIC_90_, 0.06 and 0.008 μg/ml for U.S. and EU isolates, respectively) (data not shown). Delafloxacin showed low MIC values (MIC_90_ range, 0.015 to 2 μg/ml) against Gram-positive pathogens (Table 1). Among S. aureus isolates, 98.5% of MSSA and 83.6% of MRSA were inhibited at the FDA susceptible (S) breakpoint of ≤0.25 μg/ml. Levofloxacin resistance (R) was detected in 28.1% of S. aureus isolates (7.7% of MSSA and 61.3% of MRSA): 74.6% of levofloxacin-R isolates (80.8% of levofloxacin-R MSSA and 73.3% of levofloxacin-R MRSA) were S to delafloxacin (Table 1). Whereas delafloxacin showed decreased susceptibility rates among S. haemolyticus isolates (44.4% S at ≤0.25 μg/ml; 17.8% S to levofloxacin), it was more active against S. lugdunensis (99.3% S at ≤0.25 μg/ml; 98.6% S to levofloxacin).

**TABLE 1:** Antimicrobial activity of delafloxacin tested against the main organisms and organism groups in 2014 to 2016 for ABSSSI

Organism name and group (no. of isolates)	No. of isolates (cumulative %) at MIC^*d*^ of:														MIC_50_	MIC_90_
	≤0.002	0.004	0.008	0.015	0.03	0.06	0.12	0.25	0.5	1	2	4	8	>HDT^*a*^		
Staphylococcus aureus (4,460)		2,070 (46.4)	954 (67.8)	133 (70.8)	47 (71.8)	176 (75.8)	404 (84.8)	357 (92.8)	133 (95.8)	89 (97.8)	53 (99.0)	40 (99.9)	4 (100.0)		0.008	0.25
LEVO-R (1,252)^*b*^		4 (0.3)	2 (0.5)	2 (0.6)	30 (3.0)	151 (15.1)	389 (46.2)	356 (74.6)	133 (85.2)	88 (92.3)	53 (96.5)	40 (99.7)	4 (100.0)		0.25	1
MSSA (2,766)		1,715 (62.0)	721 (88.1)	102 (91.8)	15 (92.3)	28 (93.3)	70 (95.8)	73 (98.5)	19 (99.2)	14 (99.7)	4 (99.8)	4 (>99.9)	1 (100.0)		≤0.004	0.015
LEVO-R (213)		3 (1.4)	1 (1.9)	0 (1.9)	8 (5.6)	21 (15.5)	66 (46.5)	73 (80.8)	19 (89.7)	13 (95.8)	4 (97.7)	4 (99.5)	1 (100.0)		0.25	1
MRSA (1,694)		355 (21.0)	233 (34.7)	31 (36.5)	32 (38.4)	148 (47.2)	334 (66.9)	284 (83.6)	114 (90.4)	75 (94.8)	49 (97.7)	36 (99.8)	3 (100.0)		0.12	0.5
LEVO-R (1,039)	0 (0.0)	1 (0.1)	1 (0.2)	2 (0.4)	22 (2.5)	130 (15.0)	323 (46.1)	283 (73.3)	114 (84.3)	75 (91.5)	49 (96.2)	36 (99.7)	3 (100.0)		0.25	1
Staphylococcus haemolyticus (45)		4 (8.9)	4 (17.8)	0 (17.8)	0 (17.8)	0 (17.8)	2 (22.2)	10 (44.4)	20 (88.9)	4 (97.8)	0 (97.8)			1 (100.0)	0.5	1
LEVO-R (37)						0 (0.0)	2 (5.4)	10 (32.4)	20 (86.5)	4 (97.3)	0 (97.3)			1 (100.0)	0.5	1
Staphylococcus lugdunensis (145)		6 (4.1)	38 (30.3)	85 (89.0)	14 (98.6)	0 (98.6)	1 (99.3)	0 (99.3)	0 (99.3)	1 (100.0)					0.015	0.03
LEVO-R (2)						0 (0.0)	1 (50.0)	0 (50.0)	0 (50.0)	1 (100.0)					0.12	
Streptococcus pyogenes (883)		76 (8.6)	354 (48.7)	324 (85.4)	121 (99.1)	8 (100.0)									0.015	0.03
LEVO-R (3)		1 (33.3)	0 (33.3)	1 (66.7)	1 (100.0)										0.015	
Streptococcus agalactiae (321)		10 (3.1)	67 (24.0)	166 (75.7)	71 (97.8)	3 (98.8)	0 (98.8)	1 (99.1)	2 (99.7)	1 (100.0)					0.015	0.03
LEVO-R (5)					0 (0.0)	1 (20.0)	0 (20.0)	1 (40.0)	2 (80.0)	1 (100.0)					0.5	
Streptococcus anginosus group (133)		59 (44.4)	51 (82.7)	22 (99.2)	1 (100.0)										0.008	0.015
Streptococcus anginosus (75)		32 (42.7)	30 (82.7)	12 (98.7)	1 (100.0)										0.008	0.015
Streptococcus constellatus (26)		14 (53.8)	9 (88.5)	3 (100.0)											≤0.004	0.015
Streptococcus intermedius (16)		7 (43.8)	7 (87.5)	2 (100.0)											0.008	0.015
Enterococcus faecalis (411)		1 (0.2)	1 (0.5)	0 (0.5)	18 (4.9)	102 (29.7)	150 (66.2)	43 (76.6)	21 (81.8)	54 (94.9)	21 (100.0)				0.12	1
LEVO-R (112)					0 (0.0)	1 (0.9)	5 (5.4)	13 (17.0)	19 (33.9)	53 (81.2)	21 (100.0)				1	2
Pseudomonas aeruginosa (506)		1 (0.2)	0 (0.2)	0 (0.2)	4 (1.0)	8 (2.6)	48 (12.1)	150 (41.7)	120 (65.4)	36 (72.5)	37 (79.8)	22 (84.2)		80 (100.0)	0.5	>4
LEVO-R (98)										0 (0.0)	4 (4.1)	14 (18.4)		80 (100.0)	>4	>4
Enterobacteriaceae (1,010)^*c*^		0 (0.0)	7 (0.3)	80 (3.9)	355 (19.8)	569 (45.3)	367 (61.7)	148 (68.3)	88 (72.3)	129 (78.1)	186 (86.4)	136 (92.5)		168 (100.0)	0.12	4
LEVO-R (227)								0 (0.0)	0 (0.0)	10 (4.4)	48 (25.6)	66 (54.6)		103 (100.0)	4	>4
Escherichia coli (509)		0 (0.0)	3 (0.6)	47 (9.8)	151 (39.5)	76 (54.4)	22 (58.7)	21 (62.9)	5 (63.9)	17 (67.2)	52 (77.4)	66 (90.4)		49 (100.0)	0.06	4
Klebsiella pneumoniae (298)			0 (0.0)	1 (0.3)	12 (4.4)	94 (35.9)	63 (57.0)	18 (63.1)	10 (66.4)	10 (69.8)	9 (72.8)	20 (79.5)		61 (100.0)	0.12	>4
Enterobacter cloacae (203)			0 (0.0)	1 (0.5)	13 (6.9)	88 (50.2)	66 (82.8)	13 (89.2)	3 (90.6)	1 (91.1)	6 (94.1)	6 (97.0)	2 (98.0)	4 (100.0)	0.06	0.5

a Greater than the highest dilution tested (HDT).

b LEVO-R, levofloxacin resistant.

c Includes E. coli, K. pneumoniae, and E. cloacae only.

d MIC values are in micrograms per liter.

Delafloxacin was more active against isolates of beta-hemolytic streptococci (MIC_90_, 0.03 μg/ml for both S. agalactiae and S. pyogenes; 98.8 and 100.0% S, respectively, at the FDA breakpoint of ≤0.06 μg/ml) and S. anginosus group (MIC_50/90_, 0.008/0.016 μg/ml; 100.0% S). The delafloxacin MIC_50/90_ values against 411 isolates of E. faecalis were 0.12/1 μg/ml, and 66.2% were S at the FDA breakpoint of ≤0.12 μg/ml (Table 1).

In contrast to the Gram-positive cocci, delafloxacin was less active against the Enterobacteriaceae (MIC_50/90_, 0.12/>2 μg/ml, 69.5% S at the FDA breakpoint of ≤0.25 μg/ml; E. coli, K. pneumoniae, and E. cloacae only) and P. aeruginosa (MIC_50/90_, 0.5/>2 μg/ml, 65.4% susceptible at the FDA breakpoint of ≤0.5 μg/ml). All levofloxacin-R clinically indicated Enterobacteriaceae and 95.6% of levofloxacin-R P. aeruginosa isolates were R to delafloxacin at their respective FDA breakpoints (MIC, ≥1 μg/ml and ≥2 μg/ml, respectively) (Table 1).

### Intermethod comparison.

**FIG 1:**
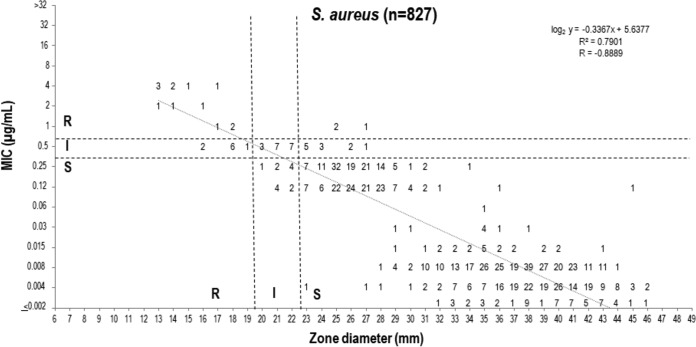
Scattergram comparing delafloxacin MIC and 5-μg disk zone diameters when testing 827 S. aureus isolates from clinical trials, surveillance surveys, and preclinical development. Broken lines indicate selected breakpoint criteria approved by the FDA (16).

**FIG 2:**
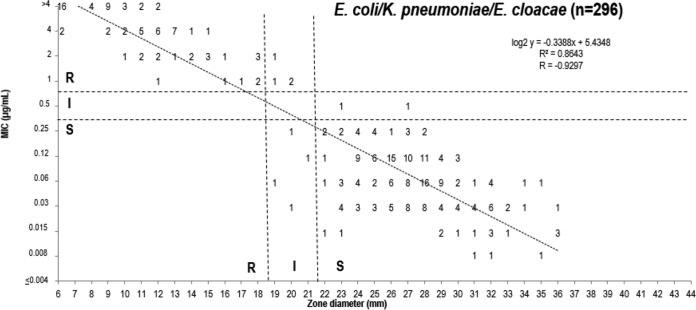
Scattergram comparing delafloxacin MIC and 5-μg disk zone diameters when testing 296 isolates from three Enterobacteriaceae species from clinical trials, surveillance surveys, and preclinical development. Broken lines indicate selected breakpoint criteria approved by the FDA (16). *n* = 58 for E. cloacae; *n* = 115 for E. coli; *n* = 123 for K. pneumoniae.

Based upon the FDA MIC breakpoints, DD method breakpoints have also been proposed for each group tested and the 5-μg delafloxacin disk content under study (Table 2). Scattergrams depicting the intermethod accuracy of the proposed MIC and DD zone diameter breakpoints are presented in Fig. 1 and 2 and Fig. S1 to S5 in the supplemental material.

**TABLE 2:** Antimicrobial susceptibility testing interpretive criteria for delafloxacin when using disk diffusion (DD) and broth microdilution (BMD) MIC methods against indicated bacterial species^*a*^

Pathogen^*a*^	BMD MIC (μg/ml)			DD zone diam (mm)		
	S	I	R	S	I	R
S. aureus (MRSA and MSSA)	≤0.25	0.5	≥1	≥23	20–22	≤19
S. haemolyticus	≤0.25	0.5	≥1	≥24	21–23	≤20
S. pyogenes	≤0.06	—	—	≥20	—	—
S. agalactiae	≤0.06	0.12	≥0.25	NC	NC	NC
S. anginosus group^*b*^	≤0.06	—	—	≥25	—	—
E. faecalis	≤0.12	0.25	≥0.5	≥21	19–20	≤18
Enterobacteriaceae^*c*^	≤0.25	0.5	≥1	≥22	19–21	≤18
P. aeruginosa	≤0.5	1	≥2	≥23	20–22	≤19

a Data from reference 16. MRSA, methicillin-resistant S. aureus; MSSA, methicillin-susceptible S. aureus; S, susceptible; I, intermediate; R, resistant; NC, no criteria; —, no criteria due to lack of clinical experience with organisms with MIC value beyond the susceptible range.

b Includes Streptococcus anginosus, S. constellatus, and S. intermedius.

c Criteria for E. coli, K. pneumoniae, and E. cloacae only.

The 5-μg disk content provided adequate separation of S and R strains of S. aureus and the Enterobacteriaceae, with rare very major error (VME; false susceptible at 0.0 to 0.4%) and no major error (ME; false resistant) for either organism group (Fig. 1 and 2). The error rates for all organism groups were well within acceptable limits (<1.5% VME and <3.0% ME) (Fig. S1 to S5). There was only one VME observed with coagulate-negative staphylococci (CoNS) (0.4%) and E. faecalis (0.9%), respectively, and none with S. pyogenes and the S. anginosus group. The overall intermethod categorical agreement (CA) ranged from 83.7% (P. aeruginosa, only 1.0% ME) to 100.0% (S. pyogenes and S. anginosus group).

### Quality control range studies.

**FIG 3:**
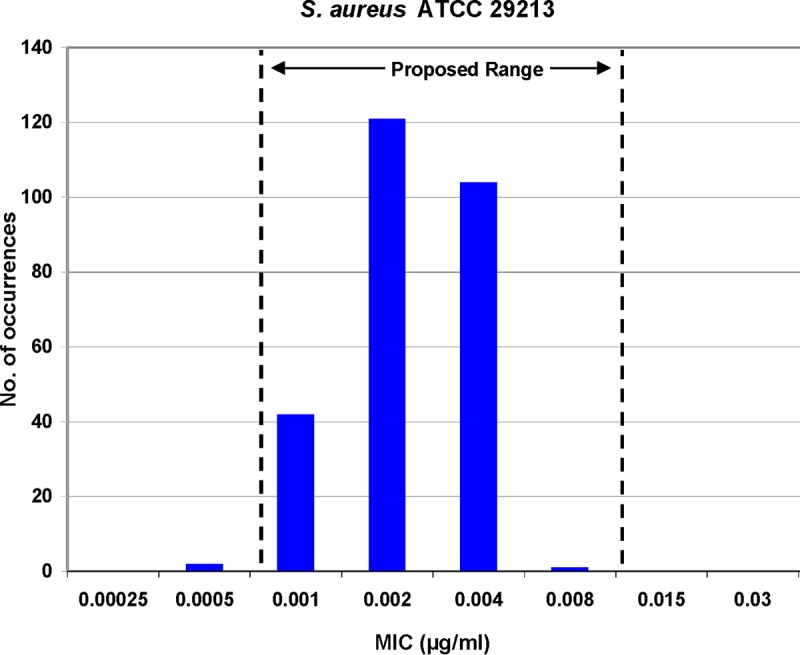
Multilaboratory (nine sites) MIC quality control study results for S. aureus ATCC 29213 tested against delafloxacin. Two hundred sixty-eight (99.3%) of 270 qualified results lie in the proposed QC range (0.001 to 0.008 μg/ml). Dashed lines indicate proposed QC limits.

The broth microdilution (BMD) method QC study had nine participating laboratories and employed reference MIC panels produced by Trek Diagnostics (Cleveland, OH). All internal FQ (levofloxacin) control results were within published ranges, validating delafloxacin data. Two QC strains required MIC ranges of four log_2_ dilution steps (S. aureus ATCC 29213 and E. faecalis ATCC 29212). A range of 0.001 to 0.008 μg/ml was calculated for S. aureus ATCC 29213 (Fig. 3), which included 99.3% of participant results with 44.8 and 38.5% of results at 0.002 and 0.004 μg/ml, respectively, a so-called “bimodal” distribution. Similarly, the E. faecalis ATCC 29212 delafloxacin MIC distribution exhibited a bimodal pattern, with 95.2% of results at either 0.03 or 0.06 μg/ml. All MIC values generated by the contributing laboratories were within the calculated E. faecalis QC range of 0.015 to 0.12 μg/ml. The remaining QC strain ranges (Table 3) had a dominant single MIC mode. The proportions of delafloxacin MIC QC study results contained in the calculated QC ranges for these strains were 96.3 to 100.0% of a total of 270 MIC values generated for each QC organism (Table 3).

**TABLE 3:** Delafloxacin susceptibility testing quality control (QC) ranges for six control strains^*a*^

QC organism	BMD MIC (μg/ml) QC range	DD zone diam (mm) QC range^*b*^
S. aureus ATCC 29213	0.001–0.008	NA
S. aureus ATCC 25923	NA	32–40
E. faecalis ATCC 29212	0.015–0.12	NA
E. coli ATCC 25922	0.008–0.03	28–35
P. aeruginosa ATCC 27853	0.12–0.5	23–29
S. pneumoniae ATCC 49619	0.004–0.015	28–36

a Results from multilaboratory study designs compliant with CLSI M23-A3 (23). Ranges approved by the FDA (16) and CLSI (20). BMD, broth microdilution; DD, disk diffusion; NA, not applicable.

b DD range using a 5-μg delafloxacin content.

QC study results for the 5-μg delafloxacin DD method were produced by two investigations utilizing zone diameters (10 laboratories) for four QC organisms. In the initial study, data from eight laboratories determined the delafloxacin zone diameter QC ranges for E. coli ATCC 25922 and P. aeruginosa ATCC 27853 calculated from 478 to 480 zone diameter results per strain using Clinical and Laboratory Standards Institute (CLSI) M23 and/or statistical criteria (Table 3). For these two tested QC strains, 96.7 to 100.0% of reported zone diameter results were within the proposed 7- to 12-mm-wide ranges. The proposed zone diameter range for S. aureus ATCC 25923 was calculated from 414 results, producing a proposed QC range between 32 and 40 mm (9 mm wide), which included 98.8% of results and a modal zone diameter at 35 and 36 mm (91 results each).

S. pneumoniae ATCC 49619 required an additional multilaboratory QC study to complete the calculated zone diameter range of 28 to 36 mm. Among 480 reported zones, 478 (99.6%) results were within the proposed range determined from 10 laboratories across two DD QC investigations. Although not a target species for ABSSSIs, S. pneumoniae is included here as an important strain for QC of delafloxacin testing of target streptococci (beta-hemolytic and viridans group streptococci).

## DISCUSSION

The *in vitro* activity of delafloxacin and support for S. aureus clinical breakpoints were presented by McCurdy et al. (10). Those data suggested a susceptible (S) breakpoint of ≤0.25 μg/ml (16) with an intermediate (I) category at 0.5 μg/ml (Table 2).

Susceptible BMD clinical breakpoints were optimized by pharmacokinetic/pharmacodynamic (PK/PD) models, Monte Carlo simulations, and target attainment (TA) of the ratio of the area under the concentration-time curve for the free, unbound fraction of the drug to the MIC (*f*AUC/MIC) for a cidal endpoint (1 log_10_ CFU reduction) (25–27). Rubino and colleagues (27) observed a 96.8 to 98.5% TA for organisms with delafloxacin MIC values at 0.25 μg/ml, and a stasis TA of 87.8 to 90.8% at 0.5 μg/ml among the modeled ABSSSI clinical trial patients treated with the approved dosing regimens (16). The S breakpoints were further adjusted for the pathogen species MIC distribution and clinical success rates indexed by organism group (16, 23–27). As an example, the percentage of eradication rates for S. aureus in phase 3 ABSSSI clinical trials with delafloxacin MIC results at 0.25 and 0.5 μg/ml were 97.2 and 100.0%, respectively (10). Furthermore, the applied *f*AUC/MIC target of 1 log_10_ CFU cidality for delafloxacin with an associated ≥90% TA was consistent with the recent breakpoint recommendations of the U.S. Committee on Antimicrobial Susceptibility Testing organization fluoroquinolone breakpoint report (28).

Delafloxacin demonstrated potent activity against S. aureus, S. lugdunensis, and the indicated streptococci (MIC_90_ results at 0.015 to 1 μg/ml) (Table 1). The levofloxacin-R strains of MRSA and S. haemolyticus tended to cluster in or near the I category for delafloxacin MIC testing results (Table 1). As reported by McCurdy et al. (10), the overall microbiological response rate was 98.6% (81/82) for all S. aureus isolates with documented quinolone resistance-determining region (QRDR) mutations from ABSSSI patients treated with delafloxacin. Notably, the delafloxacin MIC values did not increase beyond 0.5 μg/ml until at least double QRDR mutations in both *gyrA* and *parC* were observed, suggesting that delafloxacin may be useful in treating infections due to FQ-R staphylococci (10, 16).

The decreased delafloxacin susceptibility for the E. faecalis and Gram-negative species was observed among the levofloxacin-R subpopulation. However, delafloxacin is known to display enhanced activity against Gram-negative isolates, such as E. coli and K. pneumoniae, when tested in an acidic environment, which suggests that the *in vivo* activity may be increased at an infection site (1, 2, 13, 29).

The selection of correlate S DD zone diameters was made via scattergram intermethod error rate bounding analyses (23), which produced rare serious discords, with VME and ME rate ranges of 0.0 to 0.4% and 0.0 to 1.0%, respectively (Fig. 1, 2, and S1 to S5). Overall, the CA between BMD and DD method breakpoints was 83.7 to 100.0%. Only the P. aeruginosa breakpoints performed suboptimally, but the discords were dominated by minor error (15.4%) (Fig. S2). Particularly high intermethod CA rates were observed for S. pyogenes (100.0%), S. anginosus group (100.0%), Enterobacteriaceae (97.0%), CoNS (96.8%), S. aureus (95.7%), and E. faecalis (93.6%).

**FIG 4:**
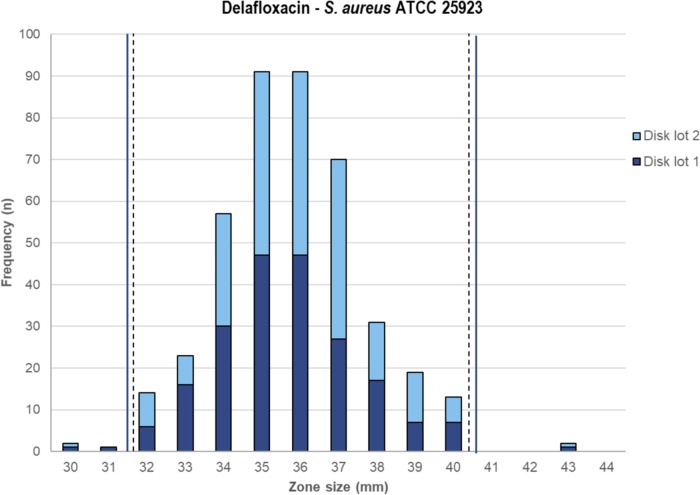
Multilaboratory delafloxacin 5-μg disk diffusion quality control study results applying three agar lots and two disk lots. Proposed range, 32 to 40 mm.

Delafloxacin antimicrobial susceptibility testing QC ranges for BMD MIC and DD methods (19, 22) have been determined for six QC strains (Table 3; Fig. 3 and 4). These QC ranges should allow this new FQ to be tested with acceptable accuracy in clinical microbiology laboratories, as these criteria are published in readily available documents (16, 20, 21).

In summary, these data extend the delafloxacin *in vitro* activity experience (11) into 2016 within a global surveillance program (Table 1) and also validate the susceptibility testing breakpoint criteria (16) for reference, standardized methods (19, 22) used by clinical microbiology laboratories. These presented results appear to be robust, derived from testing thousands of ABSSSI clinical isolates among the clinically indicated species (16), and were supplemented with the determination of QC parameters for the *in vitro* test methods (16, 20). The latter set of quality assurance guidance documents should enhance efforts to accurately assess the role of delafloxacin for treating FQ-R S. aureus and other pathogens, as well as for expanding clinical indications to respiratory tract infections (1, 11, 25, 30–32).

## Supplementary Material

Supplemental material
