# Supplementary material for: Delafloxacin In Vitro Broth Microdilution and Disk Diffusion Antimicrobial Susceptibility Testing Guidelines: Susceptibility Breakpoint Criteria and Quality Control Ranges for an Expanded-Spectrum Anionic Fluoroquinolone
Source: J Clin Microbiol. 2018 Jul 26;56(8):e00339-18. doi: 10.1128/JCM.00339-18 (PMC6062791; doi:10.1128/JCM.00339-18)
Supplement: Supplemental material [file supp_56_8_e00339-18__index.html]

Supplemental material 

# Delafloxacin *In Vitro* Broth Microdilution and Disk Diffusion Antimicrobial Susceptibility Testing Guidelines: Susceptibility Breakpoint Criteria and Quality Control Ranges for an Expanded-Spectrum Anionic Fluoroquinolone

## Supplemental material

- Supplemental file 1 -

  Fig. S1 (Scattergram comparing delafloxacin MIC and 5-μg-disk zone diameter for 283 coagulase-negative *Staphylococcus* isolates), S2 (Scattergram comparing delafloxacin MIC and 5-μg-disk zone diameter for 104 *P. aeruginosa* isolates), S3 (Scattergram comparing delafloxacin MIC and 5-μg-disk zone diameter for 82 *S. pyogenes* isolates), S4 (Scattergram comparing delafloxacin MIC and 5-μg-disk zone diameter for 134 *S. anginosus* group isolates isolates), and S5 (Scattergram comparing delafloxacin MIC and 5-μg-disk zone diameter for 110 *E. faecalis* isolates)

  PDF, 152K
