# Supplementary material for: Delafloxacin In Vitro Broth Microdilution and Disk Diffusion Antimicrobial Susceptibility Testing Guidelines: Susceptibility Breakpoint Criteria and Quality Control Ranges for an Expanded-Spectrum Anionic Fluoroquinolone
Source: J Clin Microbiol. 2018 Jul 26;56(8):e00339-18. doi: 10.1128/JCM.00339-18 (PMC6062791; doi:10.1128/JCM.00339-18)
Supplement: Supplemental material [file JCM.00339-18_zjm999096040s1.pdf]

Figure S-1. Scattergram comparing delafloxacin MIC and 5-µg disk zone diameters when testing 283 coagulase-negative staphylococci isolates (includes 25 *S. haemolyticus*) from clinical trials, surveillance surveys, and pre-clinical development.

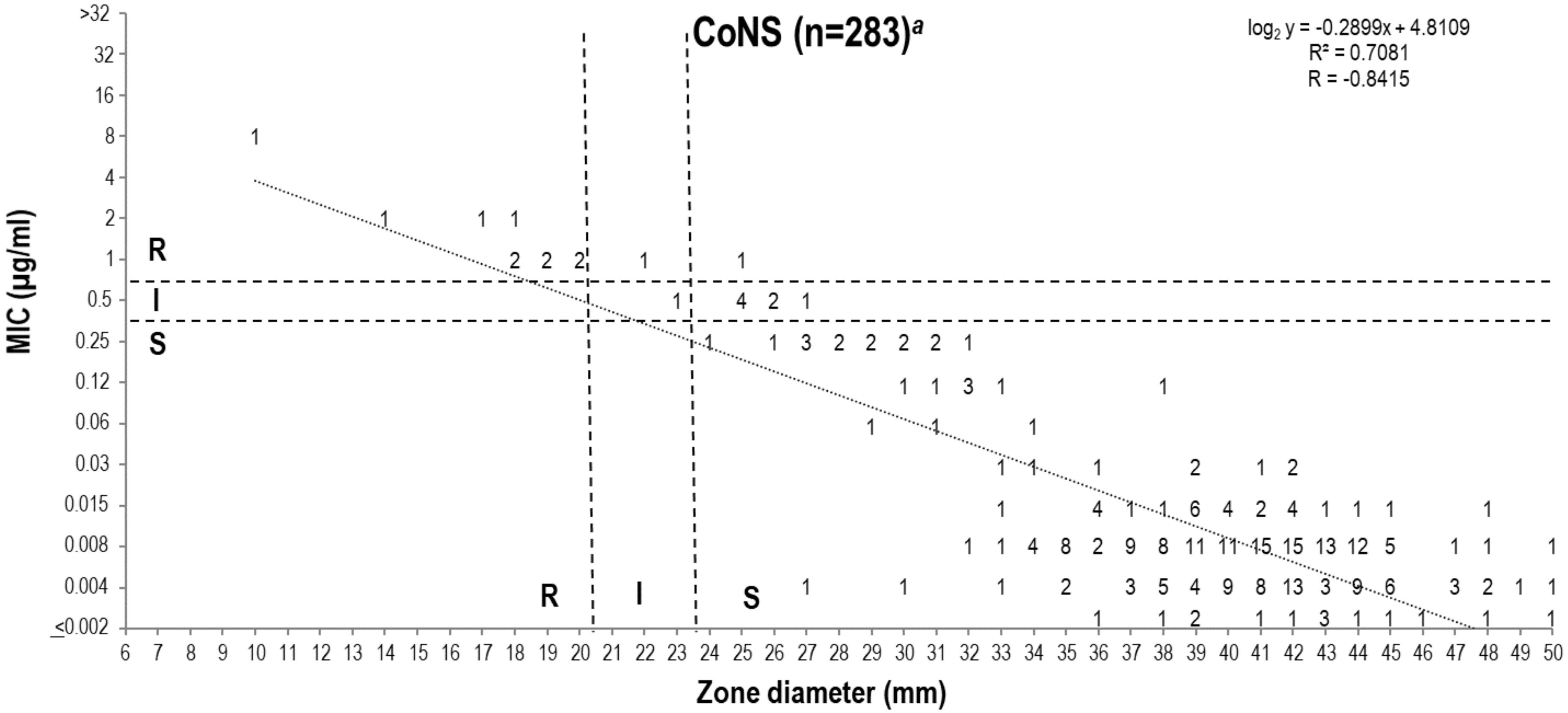

[a] Broken lines indicate selected breakpoint criteria approved by the U.S. FDA (1).

Figure S-2. Scattergram comparing delafloxacin MIC and 5-µg disk zone diameters when testing 104 *P. aeruginosa* isolates from clinical trials, surveillance surveys, and pre-clinical development.

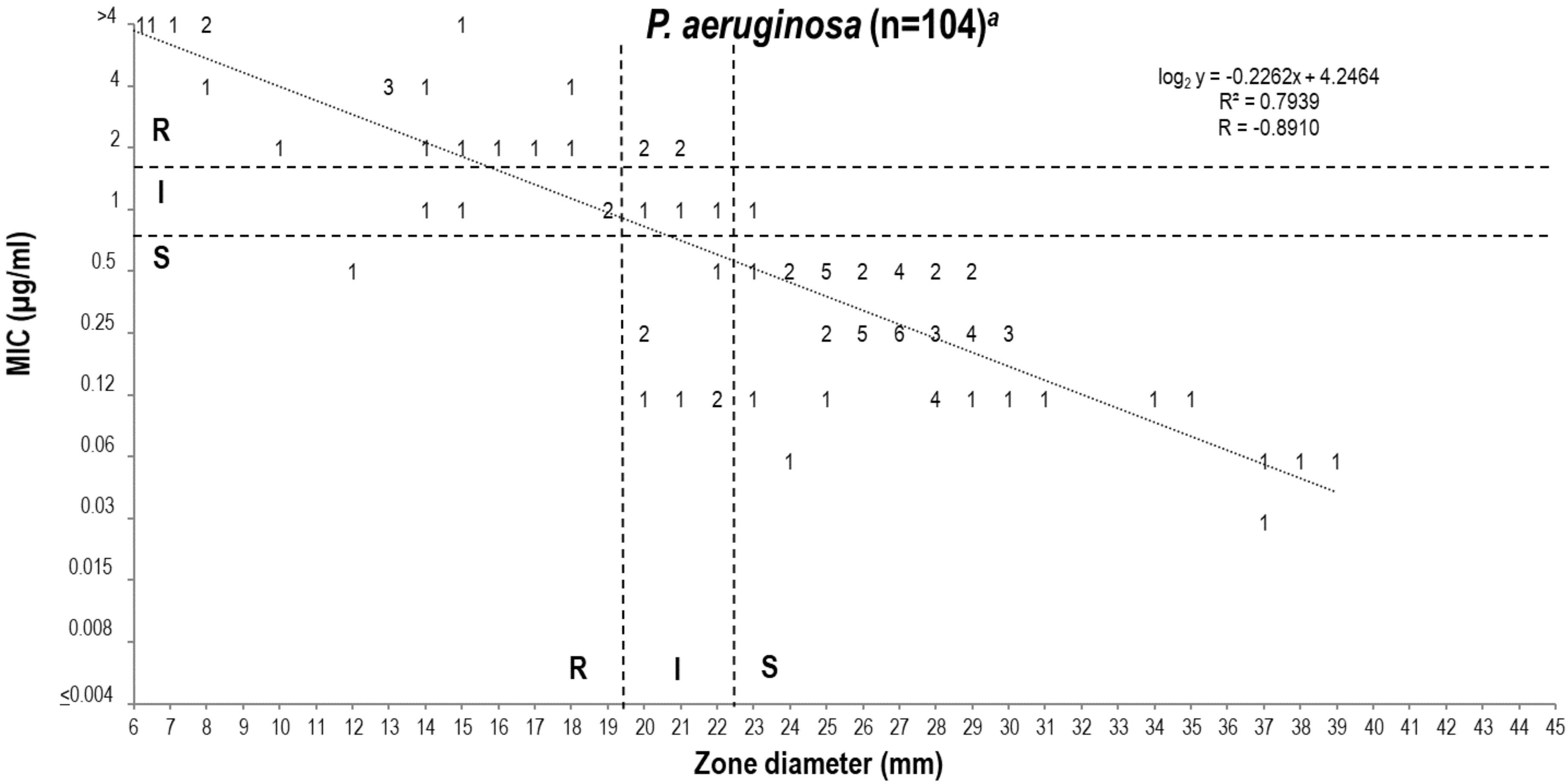

[a] Broken lines indicate selected breakpoint criteria approved by the U.S. FDA (1).

Figure S-3. Scattergram comparing delafloxacin MIC and 5-µg disk zone diameters when testing 82 *S. pyogenes* isolates from clinical trials, surveillance surveys, and pre-clinical development.

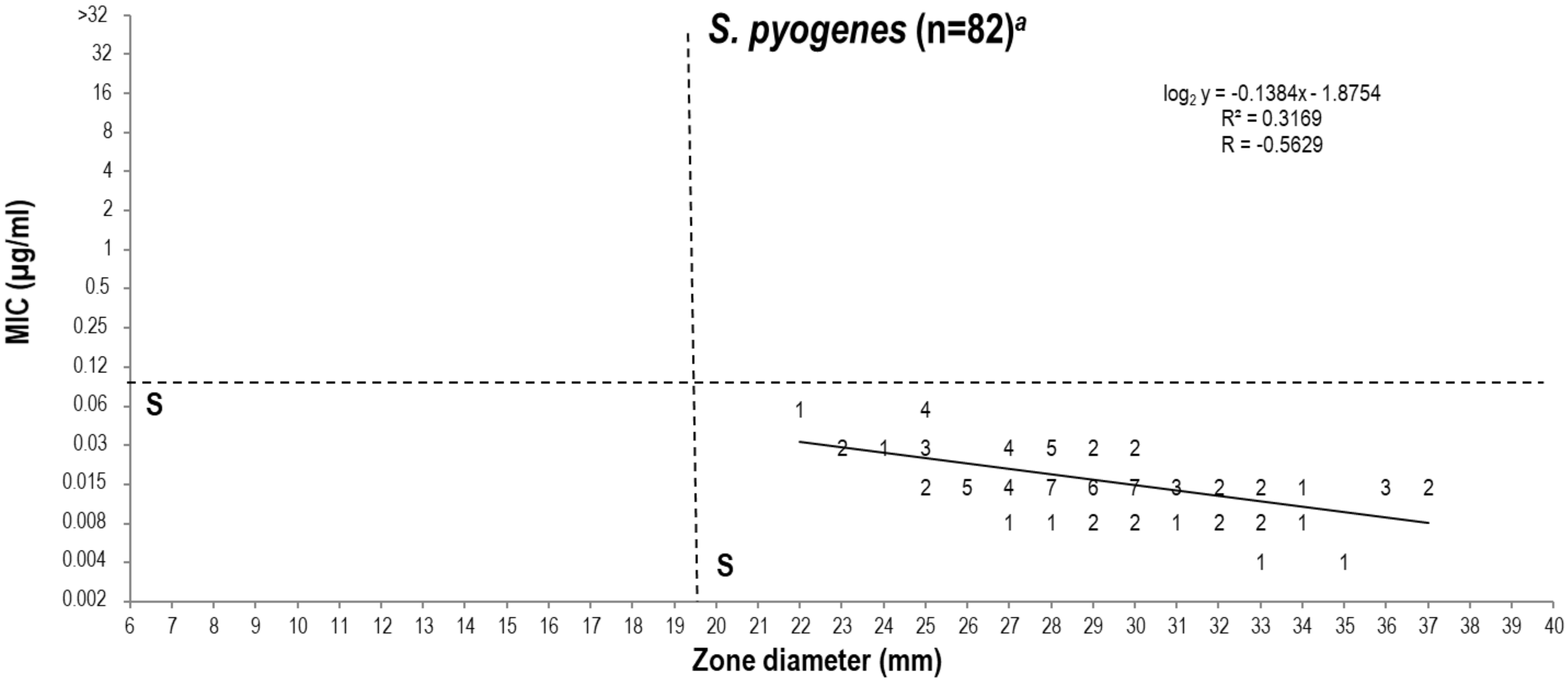

[a] Broken lines indicate selected breakpoint criteria approved by the U.S. FDA (1).

Figure S-4. Scattergram comparing delafloxacin MIC and 5-µg disk zone diameters when testing 134 *S. anginosus* group isolates from clinical trials, surveillance surveys and pre-clinical development

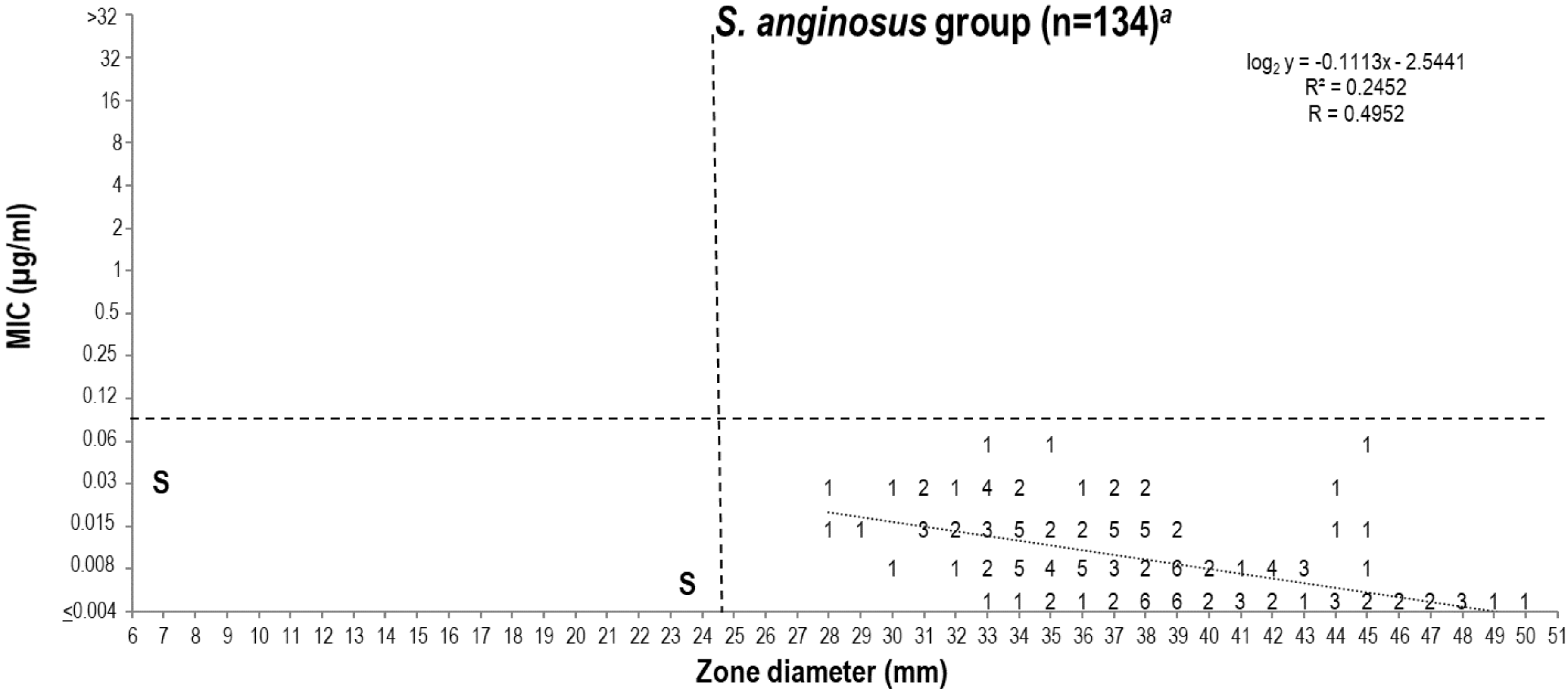

[a] Broken lines indicate selected breakpoint criteria approved by the U.S. FDA (1).

Figure S-5. Scattergrams comparing delafloxacin MIC and 5-µg disk zone diameters when testing 110 *E. faecalis* isolates from clinical trials, surveillance surveys, and pre-clinical development

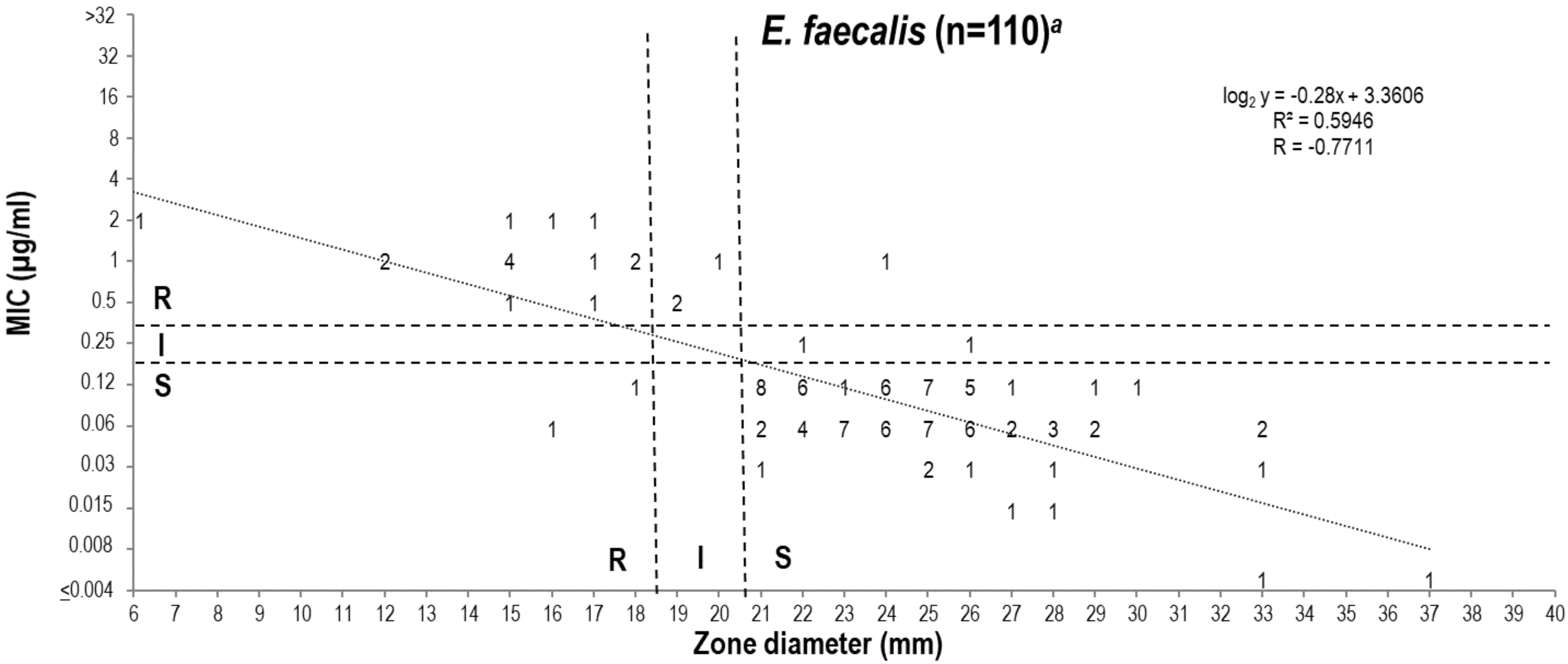

[a] Broken lines indicate selected breakpoint criteria approved by the U.S. FDA (1).

1. Baxdela. 2017. BAXDELA package insert. https://www.accessdata.fda.gov/drugsatfda_docs/label/2017/208610s000,208611s000lbl.pdf. Accessed September 2017.
